# How effective is clinical simulation in improving medical students' confidence when interviewing patients with mental health problems?: a comparison of pre-COVID and post-COVID medical student experiences

**DOI:** 10.1192/bjo.2021.728

**Published:** 2021-06-18

**Authors:** Philippa Mitchell, Ella Varnish, Arthita Das

**Affiliations:** 1 Medical undergraduate, University of Sheffield, Rotherham Doncaster and South Humber NHS Foundation Trust; 2 Rotherham Doncaster and South Humber NHS Foundation Trust

## Abstract

**Aims:**

Clinical Simulation sessions were started in April 2020 to supplement reduced patient contact for medical students at the University of Sheffield due to COVID-19 restrictions. These were run by Foundation Trainees in psychiatry with supervision and oversight from a senior psychiatrist. This study aims to review current literature on remote teaching as a learning resource and will evaluate the effectiveness of clinical simulation as an alternative to patient contact, with the focus being on improving students’ confidence as well as developing clinical interview skills.

**Method:**

Feedback surveys were developed, focussing on confidence undertaking difficult aspects of psychiatric interviews, and distributed amongst two cohorts of medical students at the University of Sheffield. One cohort completed their face-to-face psychiatry placement in full pre-COVID, the other undertook placements consisting of virtual simulation sessions alongside reduced patient contact. Responses were collected online over 6 weeks between February and March 2021. As two medical students who completed face-to-face psychiatry placement prior to the pandemic, we have additionally submitted personal reflections as a comparator to current student experiences.

**Result:**

A total of 8 students in the clinical simulation cohort, and a total of 13 students from the face-to-face teaching cohort completed the questionnaire. 62.5% of students that responded were female and the remaining percentage identified as male. Students in the face-to-face cohort reported being more confident in 6 out of 7 aspects of our feedback surveys determining confidence undertaking clinical interview skills in comparison to the virtual simulation cohort. Students attended varying numbers of simulation sessions and ultimately the main restrictions and barriers to the simulation teaching reported by students are the time constraints during the sessions, and unstable internet connection.

**Conclusion:**

Overall confidence levels in medical students are undoubtedly higher in students that completed full face-to-face placements in comparison to those with combined teaching. Based on student responses and review of current literature, clinical simulation appears to serve as a useful adjunct to students with reduced face-to-face contact in psychiatry, particularly for increasing confidence when interviewing more challenging patients. Immediate facilitator feedback and exposure to more difficult patient scenarios seem to be the most beneficial aspects. We would not advocate it as an exclusive form of teaching for medical students, but it may be a useful resource post-pandemic for providing students with extra learning opportunities, specifically targeted at developing confidence and skills in more difficult situations which will hopefully benefit them in their later careers.

